# Co‐endemicity of schistosomiasis and tegumentary leishmaniasis: Spatial co‐clustering in endemic areas

**DOI:** 10.1111/tmi.14118

**Published:** 2025-04-27

**Authors:** Genil Mororó Araújo Camelo, Jeferson Kelvin Alves de Oliveira Silva, Dharliton Soares Gomes, Laura Maggi, Stefan Michael Geiger, David Soeiro Barbosa, Deborah Aparecida Negrão‐Corrêa

**Affiliations:** ^1^ Departamento de Parasitologia, Instituto de Ciências Biológicas Universidade Federal de Minas Gerais Belo Horizonte MG Brazil

**Keywords:** bivariate LISA analysis, co‐infection, *Leishmania*, neglected tropical diseases, *Schistosoma*, spatial correlation

## Abstract

**Objectives:**

Schistosomiasis and tegumentary leishmaniasis simultaneously affect areas in tropical and subtropical regions. Co‐infected individuals show a less‐than‐optimal response to treatment and increased regulatory immune responses. However, no study has determined where *Schistosoma*–*Leishmania* co‐infections are more likely to occur.

**Methods:**

Data from The Global Health Observatory were collected to determine the worldwide endemicity status of schistosomiasis and tegumentary leishmaniasis in 2023. To determine co‐endemic areas at a local level, an ecological study was conducted on confirmed cases of American tegumentary leishmaniasis and schistosomiasis in the State of Minas Gerais, Brazil, between 2013 and 2017. Local Indicators of Spatial Association analyses were used to search for co‐endemic hotspots.

**Results:**

Thirty‐one countries were considered co‐endemic, 23 of which presented active transmission of both diseases. Univariate Local Indicators of Spatial Association indicated 13 municipalities as high–high clusters for both American tegumentary leishmaniasis and schistosomiasis in Minas Gerais. Furthermore, bivariate Local Indicators of Spatial Association analyses identified 61 municipalities as high–high clusters, grouped in seven co‐endemic hotspots.

**Conclusion:**

Local Indicators of Spatial Association analyses are a useful tool for identifying areas where co‐infection cases are more likely to occur. Similar analyses will assist authorities and healthcare providers when formulating policies and treating *Schistosoma*–*Leishmania* co‐infected patients and will provide valuable data to enable researchers to explore the impact of this and other co‐infections.

## INTRODUCTION

The World Health Organisation (WHO) classifies 20 diseases as Neglected Tropical Diseases (NTDs) for their impact on impoverished communities [1, 2]. Prevalent in tropical and subtropical regions, NTDs thrive in populations that are exposed to pathogens and lack proper sanitation and healthcare [1, 2]. Among these, schistosomiasis and leishmaniasis stand out as being two of the most debilitating NTDs.

Schistosomiasis is caused by the trematode *Schistosoma* spp. and is endemic to 78 countries, causing 2.5 million disability‐adjusted life years in 2016 [3, 4]. Leishmaniasis, caused by the intracellular protozoan *Leishmania* spp., is divided into visceral manifestations (visceral leishmaniasis) and skin and mucosal manifestations (tegumentary leishmaniasis). Tegumentary leishmaniasis is endemic to 90 countries, and 205,986 cases were reported in 2022 worldwide [1, 5].

Co‐infections are common when people are exposed to multiple pathogens, potentially altering the course of each disease, even to the point of jeopardising prognostics [6]. Indeed, virtually all protozoan‐helminth co‐infection models revealed interactions between parasites [7]. In this respect, helminth co‐infections are regarded as one of the main immunomodulatory factors that can impact the outcome of different manifestations of leishmaniasis [8].

The transmission patterns of schistosomiasis and tegumentary leishmaniasis are mostly focal [9, 10, 11, 12]. However, some shared risk factors, such as the absence of appropriated sanitation and water supply, temperature, and humidity, increase host exposure and favour co‐infections [13, 14, 15, 16]. Moreover, both diseases have immunopathogenic etiologies [17, 18, 19], and *Schistosoma* spp. infections have a high potential of affecting infectious and chronic inflammatory diseases [20, 21, 22, 23, 24, 25]. Studies analysing the impact of *Schistosoma* spp. co‐infections on American tegumentary leishmaniasis (ATL), although scarce, have shown that co‐infected individuals may present poor responses to antimony treatment and increased susceptibility to *Leishmania braziliensis* infection [26, 27, 28, 29, 30]. The mechanisms involved in those changes, recently revised [31], have yet to be further explored. However, increased serum IgE levels in individuals with helminth co‐infections, including *Schistosoma mansoni*, suggest the induction of systemic type‐2 responses [28]. In addition, ex vivo experiments showed that regulatory immune responses may also be triggered in *S. mansoni‐*co‐infected individuals. By inhibiting effective T‐cell activation via downregulation of IL‐12p40 secretion by dendritic cells and expansion of CTLA4^+^ and Foxp3^+^ helper T lymphocytes, accompanied by increased expression of IL10R and IL‐10 in dendritic cells, *S. mansoni* co‐infection could compromise the ability to control *Leishmania* spp. [32, 33, 34, 35, 36]. Importantly, Miranda et al. [37] showed that *Leishmania* spp. infections can modulate immune response against *S. mansoni* even after clinical cure of ATL.

In the Americas, Brazil is the country that bears most cases of schistosomiasis and leishmaniasis [3, 5]. Currently, Brazil is also the country that has reported the highest number of cases of *Schistosoma*–*Leishmania* co‐infections, which have been shown to occur in four different states: Bahia, Rio de Janeiro, São Paulo, and Minas Gerais [28, 29, 30, 37, 38]. Community‐based studies in co‐endemic areas in Brazil reported that the prevalence of *S. mansoni*‐*Leishmania* spp. co‐infections was as high as 16.7% in patients with ATL, while 54.8% of individuals with a history of ATL presented active schistosomiasis [26, 27, 28, 30, 37]. However, despite reports of human populations being exposed simultaneously to both diseases, areas where *Schistosoma*–*Leishmania* co‐infections are more likely to occur are yet to be determined.

Spatial analyses have previously been used to determine priority areas for surveillance and control of both schistosomiasis and ATL in Brazil, albeit separately, and they suggested that some regions of interest for each disease overlap, especially in Northeastern and Southeastern Brazil [39, 40, 41, 42, 43, 44]. Although spatial studies that indicate areas of high transmission of a single disease (either schistosomiasis or ATL) can be interpreted together to hint at possible regions of mutual transmission, integrated analyses may provide more credible evidence of co‐endemicity. However, to the best of our knowledge, no previous work has addressed the distribution of schistosomiasis and ATL in relation to each other.

Considering the impact that these diseases can have on each other and the lack of epidemiological studies addressing *Schistosoma–Leishmania* co‐infections, this study identified countries with active transmission of both schistosomiasis and tegumentary leishmaniasis. Finally, spatial analysis was applied to define the geographic distribution of highly co‐endemic areas in a region with historical transmission of both diseases in Brazil [37, 42, 45, 46], thus providing a better understanding of where co‐infections may be more likely to occur.

## MATERIALS AND METHODS

### Ethics

Ethical approval was not required since this study relied on data derived from secondary public databases. No data used herein allows the identification of individuals. The authors had no contact with any individual whose data was used; therefore, no informed consent was requested.

### Worldwide co‐endemicity

Data on the endemicity of schistosomiasis and tegumentary leishmaniasis worldwide in 2023 were acquired from The Global Health Observatory (TGHO). For schistosomiasis, endemic countries were divided into ‘requiring preventive chemotherapy’, which were considered as having active transmission of schistosomiasis; while ‘not requiring preventive chemotherapy’ and ‘status of transmission to be determined’, which were considered endemic without active transmission of schistosomiasis. Countries classified by the TGHO as ‘non‐endemic’ and ‘interruption of transmission to be confirmed’ were considered non‐endemic for schistosomiasis in our analysis. For tegumentary leishmaniasis, countries were considered as having active transmission if classified as ‘endemic’ on the database, while ‘non‐endemic’ and ‘previously reported cases’ countries were considered non‐endemic. To be considered co‐endemic, a territory must have been listed as endemic for both schistosomiasis and tegumentary leishmaniasis in 2023 according to our classification. A co‐endemicity map was created using QGIS version 3.22.3.

### Identifying concomitant transmission hotspots in an endemic region

#### Study area

**FIGURE 1:**
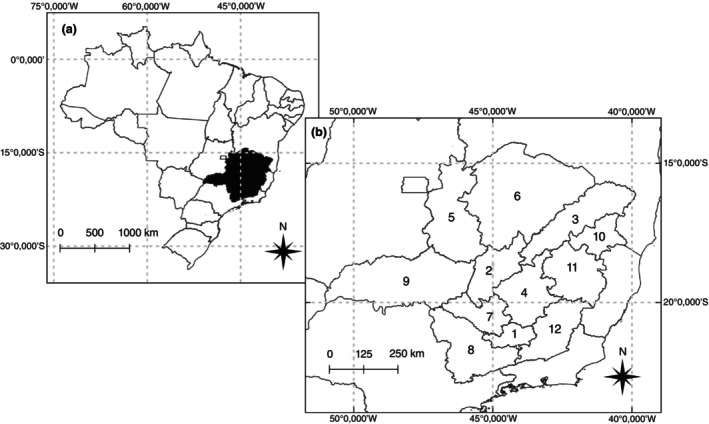
Map of the State of Minas Gerais, Brazil. (a) Map of Brazil and its federal units, with Minas Gerais in black. (b) Map of Minas Gerais showing its mesoregions. 1: Campos das Vertentes. 2: Central Mineira. 3: Jequitinhonha. 4: Metropolitana de Belo Horizonte. 5: Noroeste de Minas. 6: Norte de Minas. 7: Oeste de Minas. 8: Sul de Minas. 9: Triângulo Mineiro. 10: Vale do Mucuri. 11: Vale do Rio Doce. 12: Zona da Mata.

The State of Minas Gerais, Brazil, was selected for determining clusters of transmission of both diseases because it is historically endemic for both schistosomiasis and ATL [37, 42, 45, 46]. The state has an area of 586,528 km^2^, with 853 municipalities divided into 12 mesoregions (Figure 1).

#### Epidemiological design and data sources

An ecological study was conducted on confirmed cases of ATL and schistosomiasis in Minas Gerais reported in official databases between 2013 and 2017. Autochthonous cases of ATL were retrieved from the database of the Information System for Notifiable Diseases of the Brazilian Ministry of Health (SINAN), while cases of schistosomiasis were recovered from both SINAN, in which severe cases and cases from nonendemic areas are reported, and the Information System of the Schistosomiasis Surveillance and Control Program (SISPCE), in which non‐severe cases from endemic areas are reported [47]. For the current study, each municipality was defined as an analytical unit, and only autochthonous cases were considered. Population data for each municipality were obtained from the 2010 National Population Census, available from the Brazilian Institute of Geography and Statistics (IBGE) via the IBGE System of Automatic Recovery (SIDRA). All shapefiles used are publicly accessible on the IBGE Map Portal. The cumulative mean incidence rates (ATL) and cumulative mean prevalence rates (schistosomiasis) per 100,000 inhabitants were calculated for each municipality and re‐estimated using Spatial Empirical Bayesian smoothing.

#### Spatial analysis

A queen contiguity spatial weight matrix was used to determine neighbours of each municipality, using first order neighbourhood criteria. This type of matrix was chosen due to the highly irregular polygon sizes and shapes and the fact that case notification in SINAN and SISPCE does not include specific geographic location within each municipality. Analyses were performed on GeoDa, version 1.20 (GeoDa Center for Geospatial Analysis and Computation, Arizona, USA).

To verify the distribution of ATL and schistosomiasis cases, univariate Moran's Global Indices were calculated [48], which indicated whether the distribution of ATL and schistosomiasis cases was dispersed, clustered, or random in the study area. Bivariate Moran's Global Indices were calculated to examine the spatial codependency and occurrence of co‐endemic regions. Global indices vary between −1 and 1, with 1 indicating a perfectly clustered distribution, −1 indicating a perfectly dispersed distribution, and 0 indicating a perfectly random distribution. Pseudo *p*‐values were assigned based on the ratio between the actual value and how many values (random permutations and actual data combined) were equally or more closely clustered than the actual distribution, with *p‐*values ≤0.05 being considered statistically significant.

Furthermore, Local Indicators of Spatial Association (LISA) were calculated to determine the occurrence of areas co‐endemic for schistosomiasis and ATL. LISA allows the identification of analytical units where the value of one variable is positively or negatively associated with the spatial lag of the same variable (univariate analysis) or another variable (bivariate analysis) [49], indicating possible clusters of high incidence/prevalence rates (high–high), low incidence/prevalence rates (low–low), and spatial outliers (high–low and low–high). Pseudo *p*‐values were assigned, and significant clusters were established when *p* ≤ 0.05. Maps were created using QGIS, version 3.22.3.

## RESULTS

### Co‐endemicity of schistosomiasis and tegumentary leishmaniasis worldwide

**FIGURE 2:**
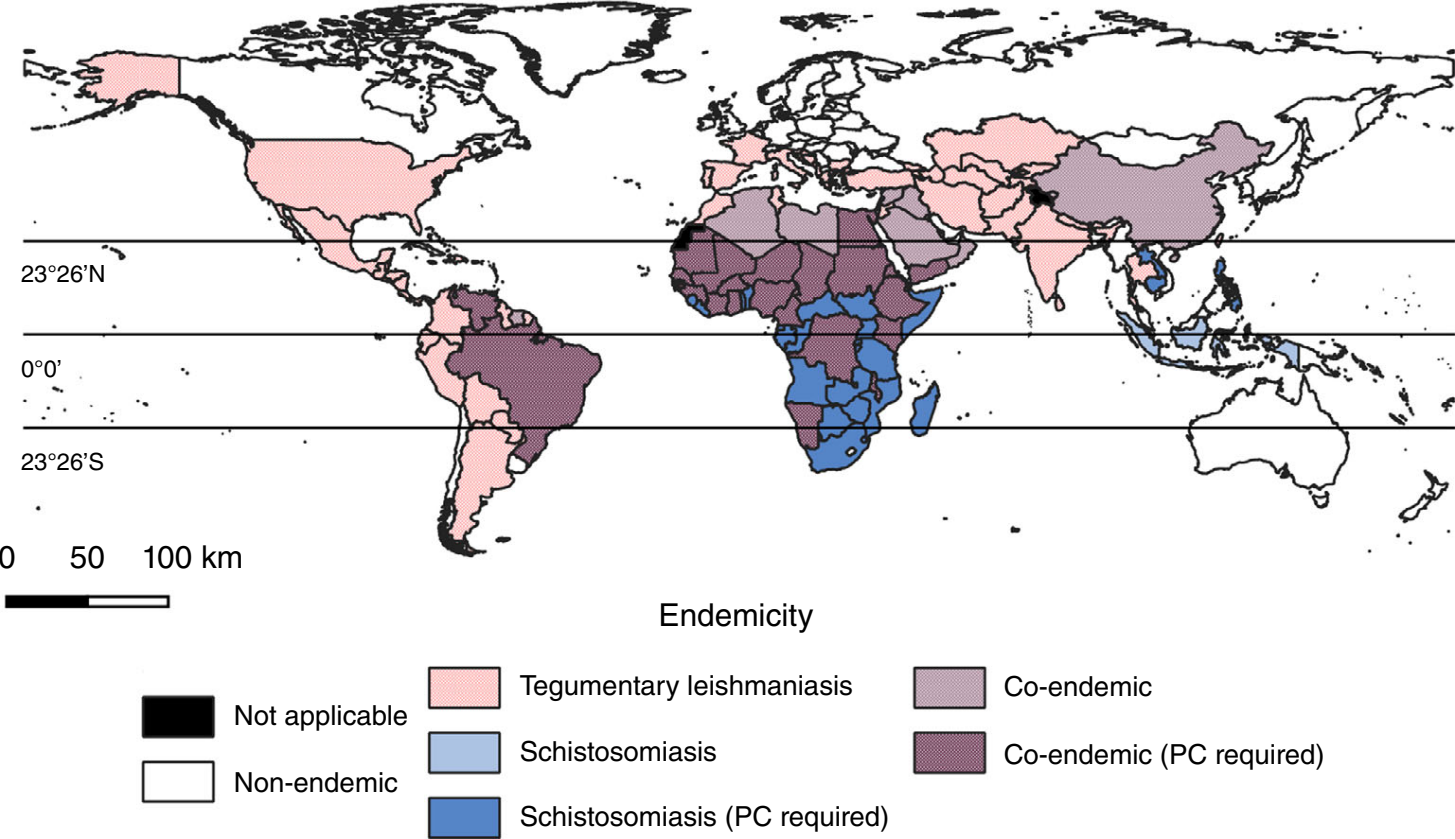
Countries co‐endemic for schistosomiasis and tegumentary leishmaniasis in 2023. PC: Preventive chemotherapy for schistosomiasis. Map created by overlapping countries endemic for schistosomiasis and countries endemic for tegumentary leishmaniasis, according to data from The Global Health Observatory.

Countries that are potentially co‐endemic for schistosomiasis and tegumentary leishmaniasis are widely distributed (Figure 2, Supplementary Table S1). Most are in tropical and subtropical regions.

Based on data from TGHO, 31 countries could be classified as co‐endemic in 2023. However, these findings should be interpreted with caution. On the one hand, while some countries classified herein as co‐endemic do not report transmission of schistosomiasis at levels that would justify the use of mass drug administration, thus decreasing the likelihood of cases of *Schistosoma–Leishmania* co‐infections, underreporting could affect one's ability to detect locations with low endemicity where this co‐infection could still have a significant impact. On the other hand, our analysis indicated that 23 countries endemic for tegumentary leishmaniasis, located in South America, Africa, and Southwest Asia, also require preventive chemotherapy for schistosomiasis, according to the WHO, deserving special attention regarding *Schistosoma–Leishmania* co‐infections.

However, it is important to consider that the transmission patterns of schistosomiasis and tegumentary leishmaniasis are usually focal [9, 10, 11, 12], which can lead to each one having differing distributions within the same country. Therefore, we used data on the prevalence of schistosomiasis and incidence of tegumentary leishmaniasis in the State of Minas Gerais, Brazil, a region regarded as endemic for both diseases, to investigate relations between the spatial distribution of such diseases more accurately.

### Co‐endemic hotspots for schistosomiasis and tegumentary leishmaniasis in Minas Gerais, Brazil

**FIGURE 3:**
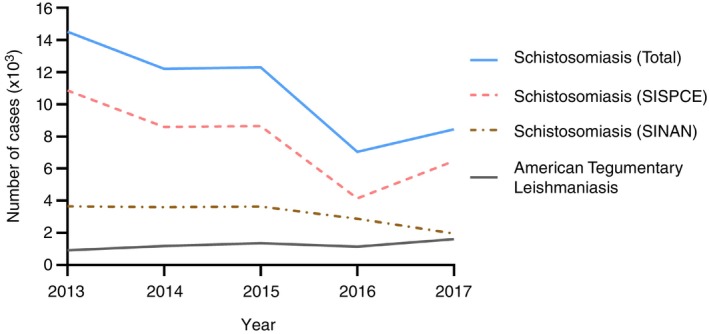
Annual notification of cases of ATL and schistosomiasis in Minas Gerais, 2013–2017. ATL cases (solid grey line) were recovered from SINAN. Total cases of schistosomiasis (solid blue line) were calculated by combining the number of reported cases in SISPCE (dashed pink line) and SINAN (dashed–dotted brown line). ATL: American tegumentary leishmaniasis. SISPCE: Information System of the Schistosomiasis Surveillance and Control Program. SINAN: Information System for Notifiable Diseases.

Between 2013 and 2017, Minas Gerais had 6241 cases of ATL and 54,496 cases of schistosomiasis, 71.1% of which were reported in SISPCE, indicating that most cases of schistosomiasis consisted of non‐severe manifestations from endemic areas. Temporal distribution of cases is shown in Figure 3. ATL cases in Minas Gerais were mostly evenly distributed from 2014 to 2017. As for schistosomiasis, reported cases presented an overall tendency for decline during the analysed period.

**FIGURE 4:**
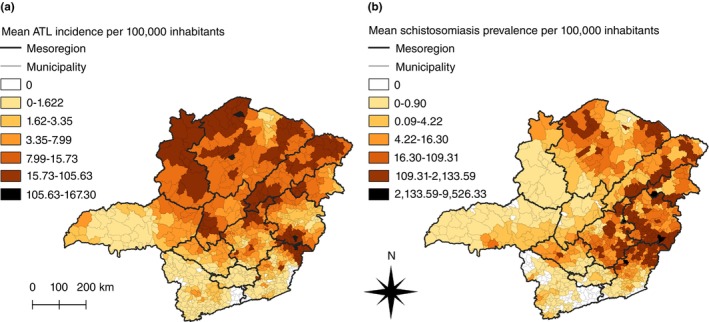
Distribution of ATL and schistosomiasis in Minas Gerais, 2013–2017. Smoothed mean incidence rates of ATL (a) and smoothed mean prevalence rates of schistosomiasis (b) per 100,000 inhabitants in Minas Gerais, Brazil, between 2013 and 2017. ATL: American tegumentary leishmaniasis.

Spatial distribution of smoothed incidence and prevalence rates is shown in Figure 4. The municipalities with the highest smoothed mean incidence rates for ATL (Figure 4a, in black) were located in the mesoregions of Norte de Minas (São João das Missões and São João do Pacuí), Zona da Mata (Simonésia), and Vale do Rio Doce (Ubaporanga and Piedade de Caratinga), while the municipalities with the highest smoothed mean prevalence rates for schistosomiasis (Figure 4b, in black) were located in the mesoregions of Vale do Rio Doce (Carmésia, Taparuba, and Ipanema), Vale do Mucuri (Franciscópolis), and Zona da Mata (Porto Firme).

The mesoregions of Triângulo Mineiro, Sul de Minas, Oeste de Minas, and Campos das Vertentes were mainly comprised of municipalities with low rates of both diseases. Noroeste de Minas and Central Mineira presented high incidence rates of ATL and low prevalence rates of schistosomiasis. In contrast, the Norte de Minas, Jequitinhonha, Vale do Mucuri, Metropolitana de Belo Horizonte, Vale do Rio Doce, and Zona da Mata were mesoregions with mostly high incidence rates of ATL and prevalence rates of schistosomiasis.

Univariate Global Moran's Index (*I*) analyses were performed to verify spatial autocorrelation, separately determining the distribution of ATL cases and schistosomiasis cases (Table 1). The distributions of ATL cases had a strong spatial autocorrelation (*I* = 0.567), whereas the autocorrelation for schistosomiasis, despite being positive, was weaker (*I* = 0.132). Meanwhile, bivariate Global Moran's Indices, which correlated ATL with schistosomiasis and vice versa (*I* = 0.127 and *I* = 0.124, respectively), showed that, although weak, there was a significant co‐dependency between the distribution of the incidence rates of ATL and prevalence of schistosomiasis (Table 1).

**TABLE 1:** Global Moran's index statistics for ATL and schistosomiasis in the Minas Gerais, Brazil, 2013–2017.

Analysis/disease	*I*	*p*‐value^a^	*z*‐value
Univariate analysis			
ATL	0.567	0.001	27.103
Schistosomiasis	0.132	0.001	7.235
Bivariate Analysis			
ATL/Schistosomiasis	0.127	0.001	8.479
Schistosomiasis/ATL	0.124	0.001	8.301

Abbreviations: ATL, American tegumentary leishmaniasis; *I*, Global Moran's index.

^a^ Pseudo *p*‐value obtained from 999 permutations.

**FIGURE 5:**
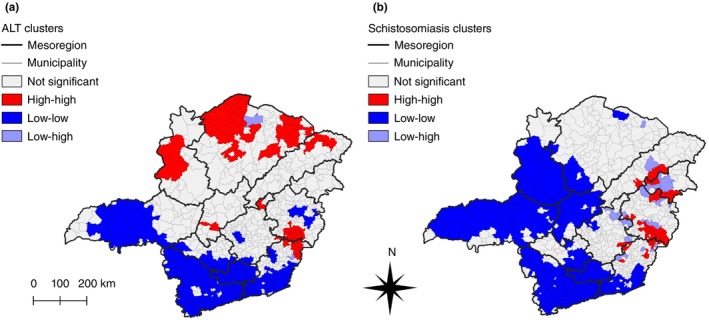
Univariate LISA cluster maps in Minas Gerais, Brazil, between 2013 and 2017. Univariate LISA analyses of smoothed incidence rates of ATL (a) and smoothed prevalence rates of schistosomiasis (b) in Minas Gerais, Brazil, between 2013 and 2017. Respectively, red and blue represent high‐high and low‐low cluster cores, while light blue represents low‐high spatial outliers. ATL: American tegumentary leishmaniasis.

To evaluate the occurrence of localised clusters with a spatial correlation between the incidence of ATL and prevalence of schistosomiasis, LISA analyses were performed (Figure 5). Figure 5a demonstrates that Triângulo Mineiro, Oeste de Minas, Sul de Minas, and Campo das Vertentes are areas with low transmission of ATL. In contrast, 66 municipalities located across Noroeste de Minas, Norte de Minas, Jequitinhonha, Central Mineira, Metropolitana de Belo Horizonte, Vale do Rio Doce, and Zona da Mata were classified as high–high clusters of ATL. In Figure 5b, which shows clusters of schistosomiasis prevalence, the municipalities in Triângulo Mineiro, Campos das Vertentes, and Sul de Minas are again clusters of mainly low prevalences; in addition, municipalities in Noroeste de Minas and Central Mineira are also mostly comprised of low–low clusters. LISA analysis revealed the existence of 41 high–high clusters of schistosomiasis in the Jequitinhonha, Vale do Mucuri, Vale do Rio Doce, Metropolitana de Belo Horizonte, and Zona da Mata mesoregions. Moreover, there were 13 overlapping high–high clusters in both analyses, in Vale do Rio Doce and Zona da Mata (Supplementary Table S2).

**FIGURE 6:**
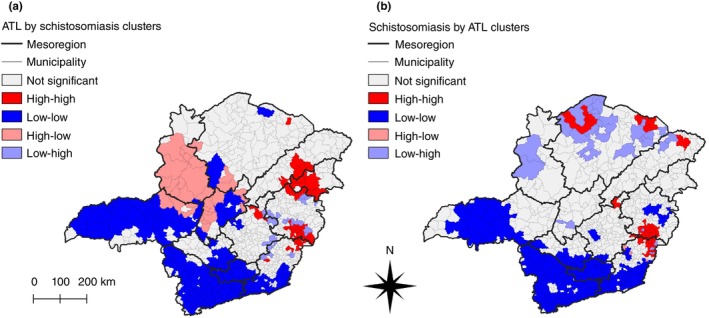
Bivariate LISA cluster maps in Minas Gerais, Brazil, between 2013 and 2017. (a) Smoothed incidence rates of ATL in a municipality in relation to the smoothed prevalence rates of schistosomiasis in its neighbourhood. (b) Smoothed prevalence rate of schistosomiasis in a municipality in relation to the smoothed incidence rates of ATL in its neighbourhood. Respectively, red and blue represent high–high and low–low cluster cores, while pink and light blue represent high–low and low–high spatial outliers.

In addition to overlapping high–high clusters, the proximity of some ATL and schistosomiasis clusters could indicate the occurrence of clusters correlating the incidence of ATL and the prevalence of schistosomiasis, with each other, in a bivariate LISA analysis. Despite the overall low bivariate Global Moran's Indices (Table 1), there were clusters of municipalities with high incidence rates of ATL with neighbouring municipalities harbouring high prevalence rates of schistosomiasis (Figure 6a), and municipalities with high prevalence rates of schistosomiasis surrounded by others with high incidence rates of ATL (Figure 6b). The bivariate LISA analyses identified 61 municipalities as high–high clusters, corresponding to 7.15% of the municipalities in the State of Minas Gerais. Of these, 40 were high–high clusters from the ATL by schistosomiasis analysis, and 34 from the schistosomiasis by ATL analysis, with 13 municipalities present in both analyses (Supplementary Table S3), the same 13 overlapping municipalities from the univariate analyses.

High–high clusters of ATL surrounded by schistosomiasis were observed in Norte de Minas, Jequitinhonha, Vale do Mucuri, Vale do Rio Doce, Metropolitana de Belo Horizonte, and Zona da Mata. Inverting the analysis to determine municipalities with high smoothed prevalence rates of schistosomiasis surrounded by neighbours with high smoothed incidence rates of ATL showed high–high clusters in the same mesoregions, except for Vale do Mucuri. The mesoregion with the highest number of high–high clusters was Vale do Rio Doce, with 19 clusters, 13 of which were municipalities in the Caratinga microregion, closely followed by Zona da Mata with 18 clusters.

**FIGURE 7:**
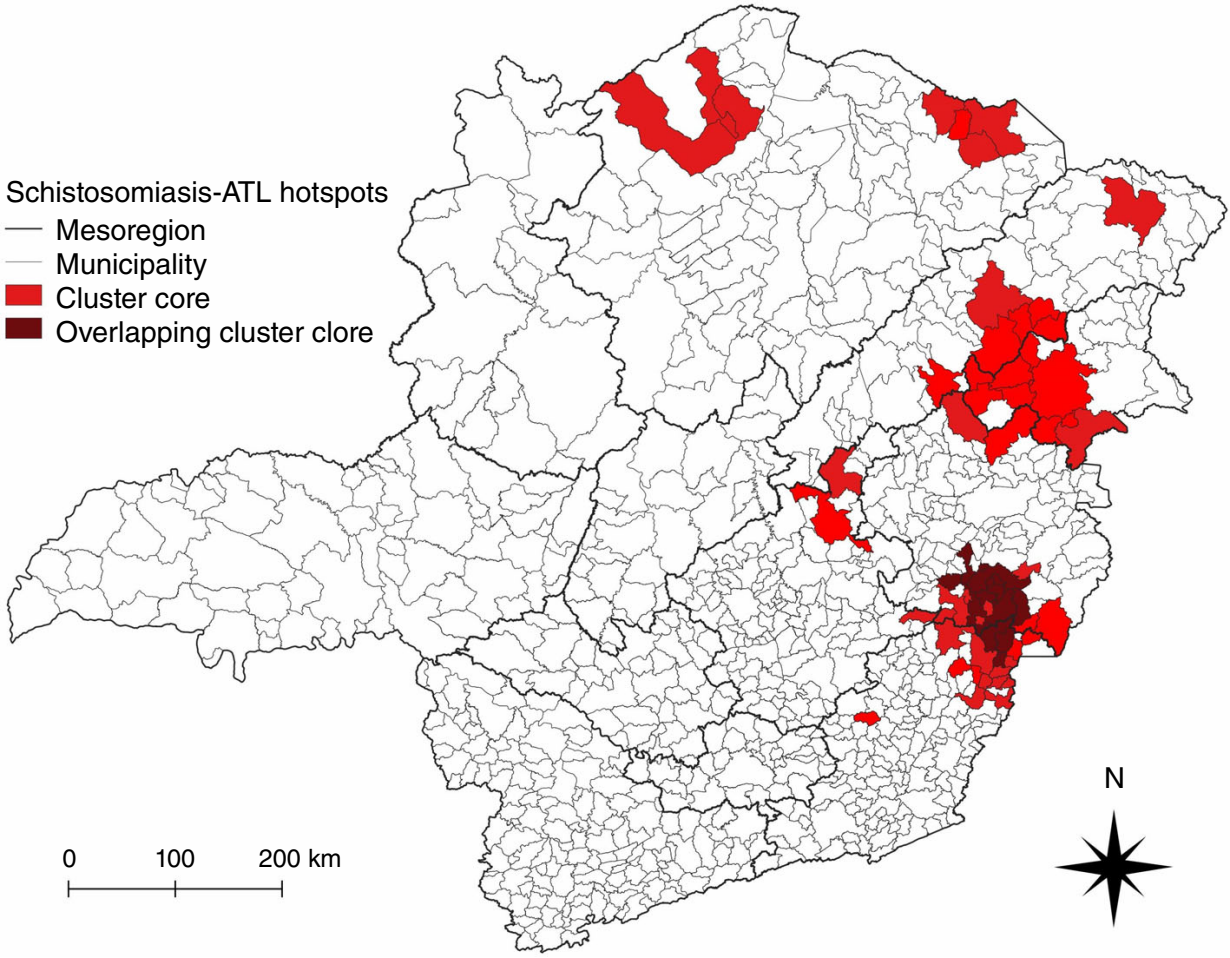
Hotspots for ATL and schistosomiasis co‐endemicity in Minas Gerais, Brazil, between 2013 and 2017. High–high clusters found in either one of the bivariate LISA analyses are presented with cores in bright red, while cores present in both bivariate analyses are shown in dark red. Map created by combining high–high clusters obtained in bivariate LISA analysis using local smoothed incidence rates of ATL by the neighbours' smoothed prevalence rates of schistosomiasis and local smoothed prevalence rate of schistosomiasis by the neighbours' smoothed incidence rates of ATL. ATL: American tegumentary leishmaniasis.

To combine the high–high clusters from both bivariate analyses, Figure 7 was constructed. Overlapping the two bivariate LISA maps revealed seven main hotspots of concomitant elevated prevalence of schistosomiasis and incidence of ATL. The smallest hotspots are in Zona da Mata and Jequitinhonha, with one municipality each. Both the hotspot in Metropolitana de Belo Horizonte and the western hotspot in Norte de Minas were composed of two municipalities each, whereas the eastern hotspot in Norte de Minas had four municipalities. The largest hotspot in area contained 16 municipalities distributed across Jequitinhonha, Vale do Mucuri, and Vale do Rio Doce. In contrast, the hotspot with the highest number of municipalities was located between the Vale do Rio Doce, Zona da Mata, and Metropolitana de Belo Horizonte mesoregions, comprising 35 municipalities, including the 13 that were identified in all LISA analyses (Supplementary Tables S2 and S3).

## DISCUSSION

Schistosomiasis and tegumentary leishmaniasis are among the most devastating NTDs. In this study, we aimed to determine which countries still had active transmission of both diseases by 2023 and can be classified as co‐endemic. In addition, we performed LISA analyses to indicate areas of co‐endemicity within a region well established as endemic for both diseases. Our study demonstrated that 31 countries are simultaneously endemic for both diseases, 23 of which are co‐endemic and classified as requiring preventive chemotherapy for schistosomiasis. Differences in control programmes across countries can impact the reliability of this finding, since their ability to detect cases may cause underreporting, while their effectiveness at reducing infection rates may quickly change the current state of endemicity.

As supported by our results, the Brazilian Schistosomiasis Surveillance and Control Program (SCP) has considerably reduced the burden of this disease, which was achieved by applying strategies based on treatment of positive cases and large‐scale treatment of at‐risk populations in endemic areas [46, 50, 51]. However, although severe cases have declined, mortality rates have remained relatively steady, which suggests flaws in notification for a plethora of factors, including but not limited to the low sensitivity of current diagnostic tools and a decreasing adherence to control activities [52, 53]. As for the effectiveness of control programmes, Deol et al. [54] assessed schistosomiasis control programmes in 10 countries, including ones that are also endemic for tegumentary leishmaniasis. While two rounds of mass drug administration were sufficient to reduce the prevalence of heavy *S. haematobium* infections in school‐aged children in Burkina Faso, Malawi, and Mali, the prevalence of heavy infections in Niger stayed above 5% [54]. Although some of these countries, such as Brazil, Burkina Faso, and Yemen, have experienced a decrease in the burden of these infectious diseases due to control programmes [5, 46, 54, 55, 56], some co‐endemic countries had their control programmes disrupted by the COVID‐19 pandemic, some of which might even be discontinued [56, 57, 58].

Moreover, even if a control programme reaches its goals, progress is unfortunately reversible, as shown in Uganda, where the prevalence of heavy *S. mansoni* infections increased 2 years after decreasing to levels below 5% [54]. This is particularly concerning in territories where mass migration and war can negatively influence the efficiency of control programmes and hinder access to basic services [59]. In addition, very little is known about how *S. haematobium* infections affect tegumentary leishmaniasis or vice versa. This calls for attention particularly because some co‐endemic countries, such as Burkina Faso and Nigeria, have most of their schistosomiasis burden caused by this species [55, 60]. It is also unknown how schistosomiasis affects cutaneous manifestations typical of non‐American leshmaniasis, such as post‐kala‐azar dermal leishmaniasis.

Despite the large number of co‐endemic countries, co‐infection between *S. mansoni* and dermotropic *Leishmania* spp. in individuals from endemic regions has only been reported in Brazil, at rates as high as 16.7% in patients with ATL [28, 29, 30, 37]. The lack of reports may not necessarily reflect a low occurrence of co‐infections but rather a lack of ability to properly investigate and record co‐infection cases. Therefore, we believe that more cases of *Schistosoma–Leishmania* co‐infections could be found worldwide if health authorities and research groups searched for them in their respective countries.

It is important to point out that, due to the focal transmission patterns of both diseases [9, 10, 11, 12], classifying an entire country as co‐endemic overestimates the problem. Thus, analyses at smaller scales can provide more accurate representations of overlapping transmission areas. Since Brazil is highly endemic for both schistosomiasis and ATL, spatial analyses were performed using the incidence and prevalence rates in Minas Gerais to identify areas where both diseases are transmitted, making co‐infections more likely to occur. The 2013–2017 timeframe was selected to avoid pandemic years, when considerable setbacks in the surveillance of NTDs happened [61, 62], and the substantial withdrawal from the Brazilian Schistosomiasis Surveillance and Control Program since 2018, which significantly impacted notification in endemic areas [53].

Historically, the State of Minas Gerais has been regarded as endemic for both schistosomiasis and ATL [40, 42, 45, 46, 63]. In addition to the reports of people who have had both diseases, albeit not concomitantly [37], Minas Gerais has had increasing incidence rates of ATL in recent years [42, 64], indicating that this state is an area where schistosomiasis‐ATL interactions are likely. In this study, Minas Gerais was used as a model to demonstrate schistosomiasis‐ATL interactions at an epidemiological level and delimit specific regions of high simultaneous incidence in a co‐endemic area.

Despite reports of *Schistosoma*–*Leishmania* co‐infections having an impact on disease development and treatment success [28, 29, 30, 37], no study has primarily addressed the epidemiological aspects of this co‐infection. To the best of our knowledge, this is the first study to spatially define areas of high occurrence for both ATL and schistosomiasis in a mutually endemic region. The results from both bivariate Global Moran's Index analyses indicate a significant, albeit weak, spatial association between the distribution of cases of schistosomiasis and ATL in Minas Gerais, indicating that the transmission of schistosomiasis and ATL may vary depending on several factors across the region. However, there are municipalities with a high prevalence rate of schistosomiasis surrounded by a neighbourhood with high incidence rates of ATL, and vice versa.

Although multifactorial, the relatively independent distribution of leishmaniasis and schistosomiasis cases in Minas Gerais can be attributed to particularities present in the parasites' life cycles and ecology. Particularly, the presence of a motile flying vector in ATL makes *Leishmania* sp. easily transmitted in peridomiciliary and domiciliary settings [65], as well as hindering the correct identification of risky behaviours of exposed individuals. In the case of schistosomiasis, transmission is necessarily associated with water contact and the presence of cercariae‐shedding intermediate hosts of the genus *Biomphalaria* [66, 67].

Moreover, the State of Minas Gerais is very diverse, both environmentally and economically [68, 69, 70], and several aspects of such diversity could impact the distribution of ATL and schistosomiasis. However, some characteristics are shared between co‐endemic hotspots. Regarding the environmental heterogeneity in Minas Gerais, most municipalities appointed as co‐endemic high–high clusters in our analyses are in regions with relatively low rainfall and high average temperatures when compared to the rest of the state, ranging from dry sub‐humid to semi‐arid climates [68]. As for social factors, it is important to mention that each municipality is autonomous and responsible for determining priorities and conducting their own disease control activities [71], which could either amplify or mitigate regional differences in the distribution of schistosomiasis and ATL. Additionally, most high–high clusters in our analyses are municipalities that presented low socioeconomic development and suboptimal supply and complexity of provided health services [69].

Spatial analyses have previously been performed to determine priority areas for the control of parasitic diseases in Brazil [39, 40, 42, 72, 73, 74, 75, 76]. The mesoregions of Norte de Minas, Vale do Mucuri, Vale do Rio Doce, Metropolitana de Belo Horizonte, and Zona da Mata, suggested as priority areas for the control of ATL and schistosomiasis [39, 40], were herein indicated as areas where cases of schistosomiasis‐ATL are more likely to occur, supporting the importance of these areas in the transmission of both diseases. The literature demonstrates that the microregion of Januária is highly endemic for both schistosomiasis and ATL [39, 42, 52, 63], and studies on schistosomiasis‐ATL interactions have been conducted there [37]. Accordingly, our findings solidify this as a favourable area for studying *Schistosoma–Leishmania* co‐infections.

In addition to concerns with the population residing in co‐endemic areas, migrations should also be considered, as there has been a report of *Schistosoma–Leishmania* co‐infection in Israel, a non‐endemic country [77]. Besides, co‐infection with one parasite may occur years after contact with the other, as both *Leishmania* spp. and *S. mansoni* can still be found years after infection [78, 79]. Moreover, urbanisation of the transmission [80, 81] could expose more people to co‐infections. Therefore, despite the local transmission of ATL and schistosomiasis [9, 10, 11, 12], it is highly possible that individuals acquire these infections separately [37].

It is also interesting to note that some clusters are located on the borders that Minas Gerais shares with the states of Bahia, where *Schistosoma–Leishmania* co‐infections have been reported [26, 27, 28], and Espírito Santo, which is also endemic for both diseases [46, 50, 82, 83]. Other neighbouring states, such as Rio de Janeiro and São Paulo, have also reported *Schistosoma–Leishmania* co‐infections [28, 29, 30]. Thus, it is important to prioritise performing similar analyses in the states of Bahia, Espírito Santo, Rio de Janeiro, and São Paulo. Additionally, similar analyses can and should be conducted in other countries, as they will be useful in raising awareness of areas where not only *Schistosoma–Leishmania* co‐infections are likely to occur but other co‐infections as well. Besides, environmental or social factors that could impact the concomitant occurrence of schistosomiasis and ATL should be the focus of future studies.

Finally, it is important to note that our study has limitations. There is an underreporting of cases of both diseases. The number of exams performed by the SCP has been decreasing since 2006, as has the number of municipalities that participate in the programme, including in the selected timeframe [53, 84]. This could have skewed our analyses towards the municipalities that still report cases in SISPCE and actively search for cases, which may have been portrayed as having higher schistosomiasis endemicity than municipalities that left the SCP. Furthermore, the current diagnostic tools for these parasitic diseases are inefficient. Standard analysis with two Kato‐Katz smears from a single stool sample substantially underestimated the prevalence of *S. mansoni* infections in low endemicity areas [52, 85, 86]. As for ATL, direct parasitological methods can be invasive and have limited sensitivity [87, 88]. Since only confirmed cases are registered, the number of cases is likely underestimated. In addition, control programmes for schistosomiasis and leishmaniasis were two of the programmes most affected by the COVID‐19 pandemic [61, 62]. Therefore, in choosing the timeframe 2013–2017 for our analyses in Minas Gerais, we aimed to avoid the pandemic years and the decline in diagnosis of schistosomiasis and leishmaniasis.

The second caveat arises because cases of schistosomiasis are registered in two separate databases [47]. This counterintuitive notification method can lead to errors, and a simplified method is required. Using both databases, we guaranteed that our analysis did not exclude any reported case of schistosomiasis. However, we cannot ensure that we are not using duplicated entries; although no case should be entered in both databases if the notification guidelines are followed properly [47].

## CONCLUSION

A noteworthy number of countries endemic for schistosomiasis or tegumentary leishmaniasis can be classified as co‐endemic. More precise spatial analysis methods, such as LISA, can assist in identifying where co‐infections are more likely to occur within these countries. In addition, bivariate LISA analyses allowed the identification of hotspots co‐endemic for schistosomiasis and ATL in the State of Minas Gerais.

We hope that identifying areas where *Schistosoma–Leishmania* co‐infections occur more frequently will assist authorities and healthcare providers to develop better control policies and appropriately assist co‐infected individuals. In addition, this study serves as a basis for future clinical studies on the impact of *Schistosoma–Leishmania* co‐infections, as determining populations with high exposure to both pathogens could possibly facilitate research groups gaining access to co‐infected individuals.

## AUTHOR CONTRIBUTIONS

GMAC, JKAOS, and DANC conceptualised and designed the study. GMAC and JKAOS collected and organised the data. GMAC, DSG, and DSB performed the analyses. GMAC, LM, and DANC wrote the manuscript. SMG, DSB, and DANC supervised the analyses and revised the manuscript. All authors read and approved the final manuscript.

## FUNDING INFORMATION

This work was supported by Fundação de Amparo à Pesquisa do Estado de Minas Gerais (FAPEMIG/Brazil) under Grant #APQ‐01637‐17 and Grant #BPD‐00829/22.

## CONFLICT OF INTEREST STATEMENT

The authors declare that they have no competing interests.

## Supporting information


Supplementary Table S1:

Supplementary Table S2:

Supplementary Table S3:

## Data Availability

Worldwide data can be accessed in The Global Health Observatory for leishmaniasis (https://www.who.int/data/gho/data/themes/topics/gho-ntd-leishmaniasis) and schistosomiasis (https://apps.who.int/neglected_diseases/ntddata/sch/sch.html). Data from Brazil can be accessed in the SINAN and IBGE databases (available at http://datasus.saude.gov.br and http://www.ibge.gov.br, respectively). Data from SISPCE in the State of Minas Gerais is provided upon request (available at https://acessoainformacao.mg.gov.br/sistema/site/index.aspx).
