# Supplementary material for: Co‐endemicity of schistosomiasis and tegumentary leishmaniasis: Spatial co‐clustering in endemic areas
Source: Trop Med Int Health. 2025 Apr 27;30(6):556–68. doi: 10.1111/tmi.14118 (PMC12136941; doi:10.1111/tmi.14118)
Supplement: Supplementary file 1 — Supplementary Table S1: [file TMI-30-556-s002.docx]

Table S1. Countries and territories co-endemic for schistosomiasis and tegumentary leishmaniasis.

| WHO Region | Country/Territory | Status of transmission* |
| --- | --- | --- |
| Africa | Algeria | No PC required |
|  | Burkina Faso | PC required |
|  | Cameroon | PC required |
|  | Chad | PC required |
|  | Côte d'Ivoire | PC required |
|  | Democratic Republic of the Congo | PC required |
|  | Egypt | PC required |
|  | Eritrea | PC required |
|  | Ethiopia | PC required |
|  | Ghana | PC required |
|  | Guinea | PC required |
|  | Guinea-Bissau | PC required |
|  | Kenya | PC required |
|  | Malawi | PC required |
|  | Mali | PC required |
|  | Mauritania | PC required |
|  | Namibia | PC required |
|  | Niger | PC required |
|  | Nigeria | PC required |
|  | Senegal | PC required |
|  | Sudan | PC required |
| Americas | Brazil | PC required |
|  | Suriname | To be determined |
|  | Venezuela | PC required |
| Eastern Mediterranean | Iraq | To be determined |
|  | Libya | To be determined |
|  | Oman | To be determined |
|  | Saudi Arabia | No PC required |
|  | Syrian Arab Republic | To be determined |
|  | Yemen | PC required |
| Western Pacific | China | No PC required |

WHO: World Health Organization. PC: Preventive chemotherapy. *Status of transmission of schistosomiasis according to The Global Health Observatory.
