# Supplementary material for: Co‐endemicity of schistosomiasis and tegumentary leishmaniasis: Spatial co‐clustering in endemic areas
Source: Trop Med Int Health. 2025 Apr 27;30(6):556–68. doi: 10.1111/tmi.14118 (PMC12136941; doi:10.1111/tmi.14118)
Supplement: Supplementary file 2 — Supplementary Table S2: [file TMI-30-556-s003.docx]

Table S2. Common high-high clusters from univariate analyses of spatial autocorrelation of ATL and autocorrelation of schistosomiasis.

| Mesoregion | Microregion | Municipality | *p*-value* |
| --- | --- | --- | --- |
| Vale do Rio Doce | Caratinga | Caratinga | 0.001 |
|  |  | Dom Cavati | 0.045 |
|  |  | Iapu | 0.048 |
|  |  | Imbé de Minas | 0.017 |
|  |  | Inhapim | 0.020 |
|  |  | São Domingo das Dores | 0.016 |
|  |  | São Sebastião do Anta | 0.021 |
|  |  | Ubaporanga | 0.019 |
|  | Aimorés | Ipanema | 0.011 |
| Zona da Mata | Manhuaçu | Reduto | 0.047 |
|  |  | Santa Bárbara do Leste | 0.036 |
|  |  | Santana do Manhuaçu | 0.014 |
|  |  | Simonésia | 0.011 |

*Pseudo *p*-value obtained from 999 permutations. The highest *p*-value from either analysis is shown.
