# Supplementary material for: Co‐endemicity of schistosomiasis and tegumentary leishmaniasis: Spatial co‐clustering in endemic areas
Source: Trop Med Int Health. 2025 Apr 27;30(6):556–68. doi: 10.1111/tmi.14118 (PMC12136941; doi:10.1111/tmi.14118)
Supplement: Supplementary file 3 — Supplementary Table S3: [file TMI-30-556-s001.docx]

Table S3. High-high clusters obtained from bivariate analyses of the spatial association between ATL and schistosomiasis.

| Mesoregion | Microregion | Municipality | Analysis | *p*-value* |
| --- | --- | --- | --- | --- |
| Norte de Minas | Januária | Cônego Marinho | S/L | 0.001 |
|  |  | Januária | S/L | 0.009 |
|  | Salinas | Indaiabira | S/L | 0.036 |
|  |  | Montezuma | S/L | 0.027 |
|  |  | São João do Paraíso | S/L | 0.050 |
|  |  | Vargem Grande do Rio Pardo | L/S | 0.027 |
| Jequitinhonha | Almenara | Almenara | S/L | 0.043 |
|  | Araçuaí | Araçuaí | L/S | 0.031 |
|  |  | Caraí | L/S | 0.031 |
|  |  | Novo Cruzeiro | L/S | 0.025 |
|  |  | Padre Paraíso | L/S | 0.046 |
|  | Capelinha | Capelinha | L/S | 0.045 |
| Vale do Mucuri | Teófilo Otôni | Ataleia | L/S | 0.022 |
|  |  | Frei Gaspar | L/S | 0.006 |
|  |  | Itaipé | L/S | 0.019 |
|  |  | Ladainha | L/S | 0.039 |
|  |  | Malacacheta | L/S | 0.007 |
|  |  | Ouro Verde de Minas | L/S | 0.028 |
|  |  | Poté | L/S | 0.007 |
|  |  | Setubinha | L/S | 0.043 |
|  |  | Teófilo Otôni | L/S | 0.021 |
| Vale do Rio Doce | Caratinga | Bom Jesus do Galho | S/L | 0.008 |
|  |  | Caratinga | Both | 0.019 |
|  |  | Córrego Novo | S/L | 0.032 |
|  |  | Dom Cavati | Both | 0.045 |
|  |  | Entre-Folhas | S/L | 0.036 |
|  |  | Iapu | Both | 0.048 |
|  |  | Imbé de Minas | Both | 0.017 |
|  |  | Inhapim | Both | 0.020 |
|  |  | Piedade de Caratinga | S/L | 0.001 |
|  |  | Santa Rita de Minas | S/L | 0.013 |
|  |  | São Domingo das Dores | Both | 0.016 |
|  |  | São Sebastião do Anta | Both | 0.021 |
|  |  | Ubaporanga | Both | 0.019 |
|  | Aimorés | Alvarenga | L/S | 0.025 |
|  |  | Conceição de Ipanema | L/S | 0.008 |
|  |  | Ipanema | Both | 0.011 |
|  |  | Mutum | L/S | 0.013 |
|  | Governador Valadares | Itambacuri | L/S | 0.014 |
|  | Peçanha | Água Boa | L/S | 0.012 |
| Metropolitana de Belo Horizonte | Conceição do Mato Dentro | Conceição do Mato Dentro | L/S | 0.002 |
|  |  | Serro | S/L | 0.047 |
|  | Itabira | Dionísio | S/L | 0.030 |
| Zona da Mata | Manhuaçu | Alto Caparaó | S/L | 0.007 |
|  |  | Alto Jequitibá | S/L | 0.003 |
|  |  | Chalé | L/S | 0.032 |
|  |  | Durandé | L/S | 0.026 |
|  |  | Luisburgo | S/L | 0.015 |
|  |  | Manhuaçu | S/L | 0.001 |
|  |  | Manhumirim | L/S | 0.038 |
|  |  | Martins Soares | S/L | 0.031 |
|  |  | Matipó | L/S | 0.030 |
|  |  | Reduto | Both | 0.047 |
|  |  | Santa Bárbara do Leste | Both | 0.036 |
|  |  | Santana do Manhuaçu | Both | 0.014 |
|  |  | São João do Munhuaçu | L/S | 0.047 |
|  |  | Simonésia | Both | 0.011 |
|  | Muriaé | Espera Feliz | S/L | 0.048 |
|  |  | Divino | S/L | 0.050 |
|  | Ponte Nova | Raul Soares | S/L | 0.004 |
|  | Viçosa | Presidente Bernardes | L/S | 0.048 |

*Pseudo *p*-value obtained from 999 permutations. In the case of a cluster present in both analyses, the highest *p*-value is shown. ATL: American tegumentary leishmaniasis. L/S: core of a high-high cluster obtained from the ATL by schistosomiasis bivariate LISA analysis. S/L: core of a high-high cluster obtained from the schistosomiasis by ATL bivariate LISA analysis. Both: core of a high-high cluster present in both analyses.
